# Fatigue Life Assessment of 65Si7 Leaf Springs: A Comparative Study

**DOI:** 10.1155/2014/607272

**Published:** 2014-10-29

**Authors:** Vinkel Kumar Arora, Gian Bhushan, M. L. Aggarwal

**Affiliations:** ^1^Department of Engineering, NIFTEM, HSIIDC, Kundli, Haryana 131028, India; ^2^Department of Mechanical Engineering, National Institute of Technology, Kurukshetra, Haryana 136019, India; ^3^Department of Mechanical Engineering, YMCA University of Science & Technology, Faridabad, Haryana 121006, India

## Abstract

The experimental fatigue life prediction of leaf springs is a time consuming process. The engineers working in the field of leaf springs always face a challenge to formulate alternate methods of fatigue life assessment. The work presented in this paper provides alternate methods for fatigue life assessment of leaf springs. A 65Si7 light commercial vehicle leaf spring is chosen for this study. The experimental fatigue life and load rate are determined on a full scale leaf spring testing machine. Four alternate methods of fatigue life assessment have been depicted. Firstly by SAE spring design manual approach the fatigue test stroke is established and by the intersection of maximum and initial stress the fatigue life is predicted. The second method constitutes a graphical method based on modified Goodman's criteria. In the third method codes are written in FORTRAN for fatigue life assessment based on analytical technique. The fourth method consists of computer aided engineering tools. The CAD model of the leaf spring has been prepared in solid works and analyzed using ANSYS. Using CAE tools, ideal type of contact and meshing elements have been proposed. The method which provides fatigue life closer to experimental value and consumes less time is suggested.

## 1. Introduction

In actual practice, the load rate and fatigue life (under specified stress range) are determined experimentally. The process of experimental fatigue life prediction of leaf springs is a time consuming process; that is, for the fatigue life of 100000 cycles, the experimental procedure will consume approximately 2-3 days. For the assessment of experimental fatigue life, a full scale leaf spring testing machine is required. The leaf spring is mounted in the machines simulating the condition of the vehicle, the fatigue test stroke is determined, and the leaf spring is tested from maximum stress to minimum or initial stress. As there are a number of factors responsible for fatigue life enhancement like material processing, loading, surface, size, and environmental factor, it is mandatory that the fatigue life should be determined by considering these factors. The engineers working in the field of leaf springs design are facing a challenge to devise a fatigue life assessment method which is reliable and consumes less time. Although the analytical or simulation techniques provide an approximate fatigue life, the validation of these results through experimental testing is mandatory. Saelem et al. [1] simulated a leaf springs model. An experimental leaf springs model was verified by using a leaf springs test rig that could measure vertical static deflection of leaf springs under static loading condition. The results showed a nonlinear relationship between the applied load and the leaf springs deflection for both directions of loading, in form of a hysteresis loop. Refngah et al. [2] worked on the possibility and capability of replacing the multileaf with the parabolic spring in suspension system. He performed the finite element analysis to analyze the stress distribution and behavior of both the springs. Then, time histories service loading data was analyzed and damage area was simulated to predict the fatigue life of the components. The simulation results are compared and validated with the experimental results. Fuentes et al. [3] studied the origin of premature failure analysis procedures, including examining the leaf spring history. The visual inspection of fractured specimens and simulation tests on real components were also performed. It was concluded that fracture occurred by a mechanism of mechanical fatigue initiated at the region of the central hole, which suffered the highest tensile stress levels. Aggarwal et al. [4] evaluated the axial fatigue strength of EN45A spring steel sample experimentally as a function of shot peening in the circumstances used. *S*/*N* curves of the samples were correlated with leaf springs curve in vehicles. Aggarwal et al. [5] concluded that influence of high contact pressure and temperatures resulted in micro weld between the two leaf surfaces. The fatigue strength of the leaf springs was studied as a function of shot peening parameters. Patunkar and Dolas [6] worked on nonlinear force displacement of each leaf spring as well as the spring characteristics of a pack consisting of two to four leaves using ANSYS. The results from ANSYS were compared with those from the test, which showed a fairly good agreement with each other. Kumar and Vijayarangan [7] described static and fatigue analysis of steel leaf springs and composite multileaf springs made up of glass fibre reinforced polymer using life data analysis. The dimensions of an existing conventional steel leaf spring of a light commercial vehicle were taken and verified by design calculations. Static analysis of 2-D model of conventional leaf springs was also performed using ANSYS 7.1 and the results obtained were compared with experimental results. Sanjurjo et al. [8] performed two peening treatments on two different surface conditions. It has been confirmed that the surface finish was the critical factor for the enhancement of the fatigue performance of the reinforced bars. It was concluded that 90% of the total fatigue enhancement is due to the removal of surface stress raisers and the enhancement of the product roughness and only 10% is due to the CRSF layer induced by shot peening. Zhuang and Halford [9] proposed a model based on the bauschinger effect and a realistic back-stress based plasticity stress-strain relation. The model was verified using an advanced finite element model. The analytical model was used to predict the residual stress relaxation versus various cyclic loading conditions. Effect of load ratio on residual stress relaxation was also depicted. He suggested that although the analytical model proposed here is able to predict the trends of residual stress relaxation, an experimental study on cycle-dependent residual stress relaxation is also required. Savaidis et al. [10] developed a finite element base model of high strength and high performance leaf springs. He described the mechanical behavior of the leaf spring under damaging driving maneuvers. The ideal type of meshing elements and contacts in the leave thickness is considered to describe the mechanical behavior in an accurate and time effective manner. Experimental results for a serial front axle multileaf spring, subjected to vertical and braking loads, were compared and validated with numerically determined stress distributions.

The objective of the present work is to provide a method for precise fatigue life assessment of leaf springs rather than experimental testing. A LCV leaf spring is taken into consideration for this study. The experimental fatigue life and load deflection are determined on a full scale leaf spring testing machine. The loading conditions for the required fatigue life are specified by the vehicle manufacturer. Four numbers of specimens with specified geometrical parameters and similar material processing are manufactured and tested for fatigue life. The alternate fatigue life assessment method comprises graphical method, analytical method (using codes in FORTRAN), SAE spring design manual method, and CAE solution (using Ansys). The fatigue life by various techniques is compared and alternate method which gives results closer to experimental is suggested.

## 2. Material

The 65Si7/SUP9 grade material is used for the experimental work. The chemical composition of the material is Mn—0.72%, C—0.53%, S—0.007%, Si—0.20%, P—0.019%, and Cr—0.73%. The heat treatment is done at 880°C and oil quench hardened and it is tempered at 410°C, for 90 minutes to get tempered martensite structure. The mechanical properties and parameters of the 65Si7 are shown in Table 1.

## 3. Leaf Spring Design Parameters

**Figure 1:**
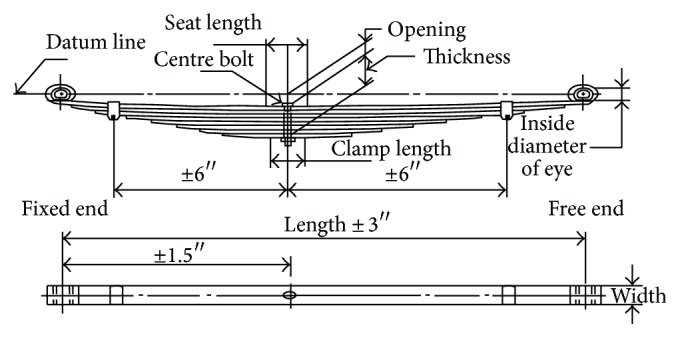
Layout drawing of the leaf spring assembly.

To design semielliptical springs, the terms like span, no load assembly camber, loaded camber, stack height, opening, and seat length are used, and these parameters are termed design parameters. The layout drawing of the leaf spring assembly is depicted in Figure 1. The line which passes through the centre of the eyes is termed datum line for the springs with eyes. Span is termed the distance between the centres of the eyes.

**Figure 2:**
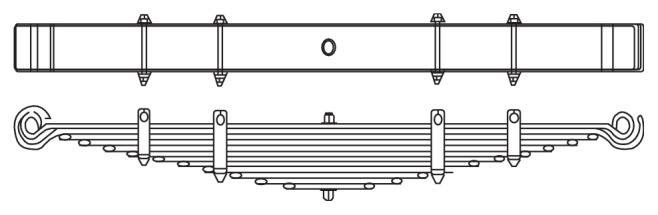
Leaf spring assembly drawing.

The distance from the datum line to the point where the centre bolt or the cup centre intersects the top surface of the main leaf when the spring is not loaded is called free camber or free height. This may be either positive or negative. The distance from the datum line to the point where the centre bolt or the cup centre intersects the top surface of the main leaf when the spring is loaded is called loaded camber. Ride clearance may be termed as the spring travel on the vehicle from the design load to the metal to metal contact position or the deflection from the design load to the metal to metal contact position. Stiffness factor takes into consideration the leave length and type of end used. The various design parameters of the leaf spring assembly of light commercial vehicle are indicated in Table 2. The layout drawing of light commercial vehicle (LCV) leaf spring is depicted in Figure 2.

### 3.1. Manufacturing of Leaf Springs

For the fabrication of high strength leaf springs, the process comprises shearing, punching, heat treatment, hot cambering, shot peening, scragging, and testing for load rate and fatigue life. After punching and shearing, the raw material is moved to the hardening furnaces for heat treatment. The structure of the raw material is partial austenite and, after quenching, the structure is martensite, but, after the tempering process, the structure should be tempered martensite. The spring steel is having the thickness of 8 mm and width of 70 mm which is heated at 880°C to achieve full austenite structure. Hot cambering of the spring is done in this state by passing through a finger cambering tools followed by quenching in oil at temperature of 80°C. Tempering is done at a temperature of 410°C for 90 min of slow cooling till the tempered martensite structure is achieved. Surface treatment like shot peening and graphite coating is done on each individual leaf. The final assembly is done by pulling all the leaf with a centre nut and bolt. The scragging of the assembly is done and the u clips are attached. The assembly is tested for load rate and fatigue life.

## 4. Experimental Setup

**Figure 3:**
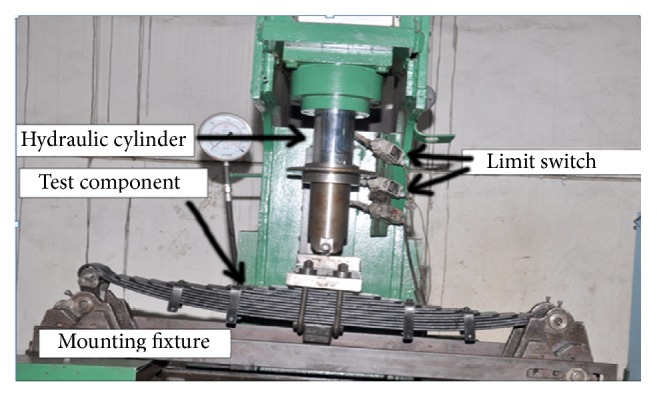
Full scale leaf spring testing machine in fully laden condition.

The 65Si7/SUP9 leaf springs assembly consists of two full length leaves and ten graduated leaves, four rebound clips of mild steel, four shim pipes with four nuts and bolts, four rivets, centre nut and bolt and bush of bronze. The master leaf consists of upturned berlin eye at both the ends. The second leaf is provided with a military wrapper to avoid accidents in case of master leaf failure at eye section. The full scale testing of leaf springs was carried out in an electrohydraulic static component testing system. The laminated leaf springs were placed in a fixture simulating the conditions of a vehicle. The setup consists of a hydraulic power pack to give a hydraulic pressure of 20.6 MPa with a flow rate of 210 liters per minute (lpm), which was sent to a hydraulic actuator to operate at a frequency of 0.3 Hz with the displacement specified by the alternating load. This involves applying the axial load on the leaf springs and measuring the deflection and bending stress. The conventional leaf spring was tested under static load condition by using hydraulic static load ram for load application. Mounting of the leaf spring was done by keeping it in inverted manner on the test bed. Two eye ends were held in the clamping devices and load was applied from the top, at the center of leaf springs. To measure the load, dial indicator was used which was located beside the full scale leaf spring testing machine and deflection was measured by strain gauges located at the clamping of the test rig. The springs were loaded from unladen load (i.e., 7.6 KN) to maximum load (i.e., 28 KN). The vertical deflection of the springs at the unladen load, design load, flat load, rubber touching load, and metal to metal contact or maximum load was recorded, respectively, as per the standard operating procedure prescribed [11].  Figure 3 shows the full scale leaf spring testing machine in static and fully laden condition.

The leaf springs were tested on a full scale leaf spring testing machine under the unladen, rated, flat, rubber touching load, and metal to metal load and the corresponding deflection and stress values observed are shown in the Table 3. The experiments were conducted twice and the mean value of the results was considered. Table 3 depicts the observed values of deflection and stress corresponding to the loads applied on the shorter leaf by a static hydraulic ram.

### 4.1. Experimental Fatigue Life Determination

To determine experimental fatigue life, four leaf springs specimens (S-1, S-2, S-3, and S-4) are manufactured with specified design parameters. Analogous kind of material processing normal rolling, quenching at 880°C, hardening in oil at 80°C, tempering at 410°C for 90 mins, shot peening at 18 A intensity, BHN 380–432, and scragging at 0.9% of yield stress was done for all the specimens. The stress range considered for the specimens is 627 MPa, 1.3 ± 0.7 g. All the four specimens were tested under same stress range and fatigue life was determined as depicted in Table 4. The fatigue life of specimen S-1 is 84212, for sample S-2 is 81961, and for samples S-3 and S-4 are 82226 and 85685, respectively. The mean value, that is, 83513 of the fatigue life for the four specimens, is taken into consideration for this study. As per the requirement specified by the vehicle manufacturer, the leaf springs are to be tested on full scale testing machine as per 1.3 ± 0.7 g. The maximum load will be 2 g and the minimum load will be 0.6 g. Here, g represents the design load [12]:

(1) Smax⁡=Pmax⁡∗La∗t8∗∑Itotal=896 MPa,Smin⁡=Pmin⁡∗La∗t8∗∑Itotal=269 MPa.

## 5. SAE Spring Design Manual Approach

Fatigue life is articulated by the number of deflection cycles that a spring will withstand without failure or permanent set. A leaf spring used in a suspension will undergo a large number of cycles of small amplitude near the design load position without failure. Under the greater amplitude, the number of cycles without failure will be reduced, since the maximum stresses as well as the stress range are amplified and both are determining factors in fatigue life of the spring. As per the SAE spring design manual approach [12], the fatigue test stroke for leaf springs (by considering assembly stress) is predicted as

- deflection at design load = 12959/153.1 = 84.6 mm,
- maximum load on the leaf springs = 28 KN,
- metal-to-metal clearance (compression stroke) = 94.6 mm,
- total deflection to maximum load = 182.9 mm,
- stress at metal-to-metal contact position = 885 MPa,
- stress rate = 885/182.9 = 4.83 MPa/mm,
- release stroke = 0.5 × 94.6 = 47.3 mm,
- fatigue test stoke = 47.3 + 94.6 = 141.9 mm,
- initial stress = 885 − (141.9 ×.4.83) = 199.6 MPa.

The maximum stress of 885 MPa and initial stress of 199.6 ≈ 200 MPa are utilized to predict the approximate fatigue life of the leaf spring.

### 5.1. Fatigue Life Assessment by SAE Spring Design Manual Approach

**Figure 4:**
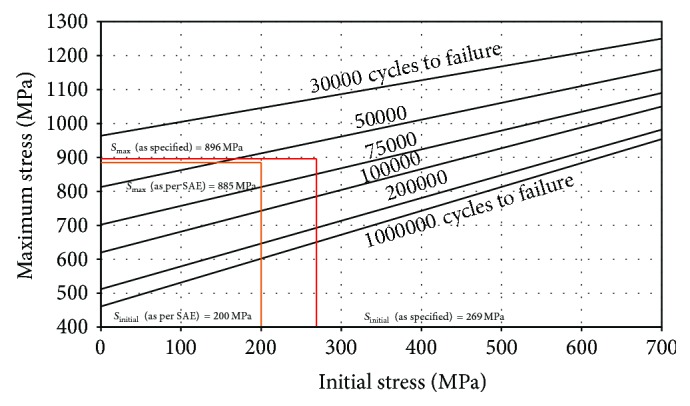
Maximum versus initial stress plot (without shot peening) [12].

Figure 4 shows a maximum stress versus initial stress plot as per SAE spring design manual approach. As per SAE spring design manual, this criterion is frequently used for determination of approximate fatigue life of the spring; initial stress (horizontal scale) and maximum stress (vertical scale) are intersected to estimate the number of cycles the spring will survive. The life predicted from this plot does not consider the effect of shot peening and scragging operations, but in actual practice processing like shot peening and scragging is performed on the leaf springs, which enhances the fatigue life by approximately 20%.

From Figure 4, it is observed that the maximum stress induced in the leaf springs (by considering the assembly stresses) is 885 MPa and the initial stress value is 200 MPa. The intersection of 885 MPa and 200 MPa lies in the zone of 50000 to 75000 cycles. As the point of intersection is nearer to 50000-cycle line, it will sustain approximately 58000 cycles. Due to shot peeing and scragging, the fatigue life of leaf springs by considering assembly stresses would be approximately (58000 ∗ 1.2)  69600 cycles.

The vehicle manufacture has specified that the maximum stress of 896 MPa corresponds to the maximum load of 2 g and the minimum stress of 269 MPa corresponds to the minimum load of 0.6 g, where g is the design load. The intersection of 896 MPa and 269 MPa lies in the zone of 50000 to 75000 cycles. As the point of intersection is nearer to 75000-cycle line, approximately it will sustain around 68000 cycles. Due to shot peeing and scragging, the fatigue life of leaf springs (by considering assembly stresses) would be approximately (68000 ∗ 1.2)  81600 cycles.

Hence, it is observed that, with the stress range obtained from SAE spring design approach, the fatigue life and as specified stress range are around 69600 cycles and 81600 cycles approximately. The stress range specified by the vehicle manufacturer gives result closer to the experimental results.

## 6. Fatigue Life Estimation by Graphical Method

**Figure 5:**
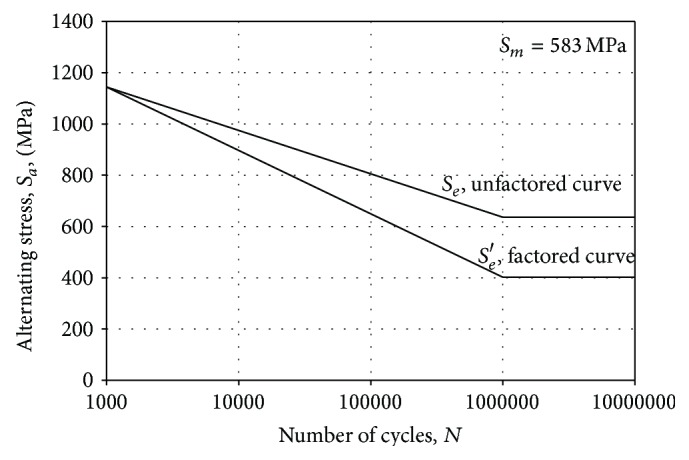
Factored and unfactored* S-N* curve for 65Si7 leaf spring.

The graphical method involves determination of effect of various factors like load, temperature, size, and surface on the endurance limit of the 65Si7.  Figure 5 shows an alternating versus number of cycles plot for unfactored endurance limit and factored endurance limit. It is observed from Figure 5 that the endurance limit is 636 MPa without considering the various factors affecting the fatigue life. It has also been observed that the corrected endurance limit is 401.9 MPa. Considering the design, material, and processing parameter of the leaf springs, the steps involved in the graphical methods are as follows:

(a) *S*_*ut*_ = 1272 MPa,
(b) *S*_*y*_ = 1081.2 MPa,
(c) *S*_*e*_′ = 0.5*S*_*ut*_ = 0.5 × 1272 = 636 MPa,
(d) *σ*_min⁡_ = 269 MPa at 0.6 g load, *σ*_max⁡_ = 897 MPa at 2 g load,
(e) *S*_*a*_ = (*σ*_max⁡_ − *σ*_min⁡_)/2 = (897 − 269)/2 = 314 MPa,
(f) *S*_*m*_ = (*σ*_max⁡_ + *σ*_min⁡_)/2 = (897 + 269)/2 = 583 MPa,
(g) *S*_*e*_ = *K*_load_ × *K*_surfae_ × *K*_temp_ × *K*_reliability_ × *K*_size_ × *S*_*e*_′,
(h) *K*_load_ = load  factor, for bending *K*_load_ = 1,
(i) *K*_surface_ = 1 (for shot peening treating),
(j) *K*_temp_ = 1; if *T* ≤ 450°C,
(k) *K*_reliability_ = 0.80, Assuming 99% reliability,
(l) *K*_size_ = 1.189∗(*d*_eqiv_)^−.097^ for 8 mm < *d* ≤ 250 mm,
(m) $d_{\text{ eqiv.}}=\sqrt{A_{95}/0.0766}=65.88$ mm,
(n) *A*_95_ = 0.05∗*b*∗*h* = 332.5 mm^2^,
(o) *K*_size_ = 1.189∗(*d*_eqiv_)^−.097^ = 0.79,
(p) *S*_*e*_ = 1 × 1 × 1 × 0.80 × 0.79 × 636 = 401.9 MPa.

**Figure 6:**
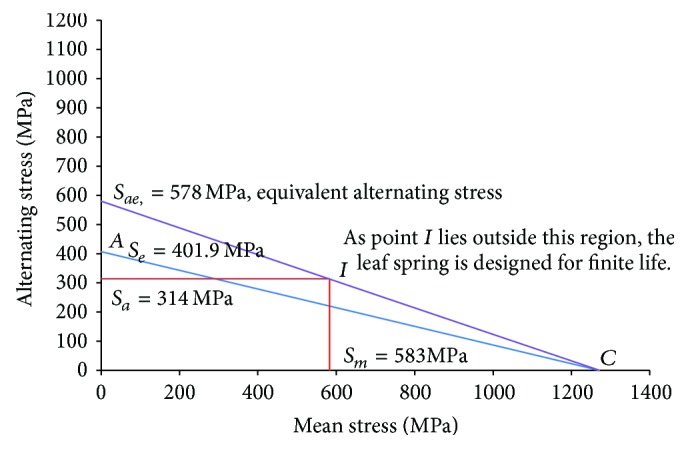
Alternating stress versus mean stress plot.

Figure 6 shows an alternating stress versus mean stress plot for the LCV leaf spring. It has been observed from Figure 6 that the intersection of the alternating and mean stress lines lies outside the region AC. If the point is within this line AC, the component is designed for infinite life but if the component is outside this region, the component is designed for the finite life. Thus, from the position of point I, it is observed that the component is designed for the finite life. The equivalent alternating stress as determined by joining the point of intersection I and ultimate strength point with the alternating stress axis is found to be 578 MPa.

**Figure 7:**
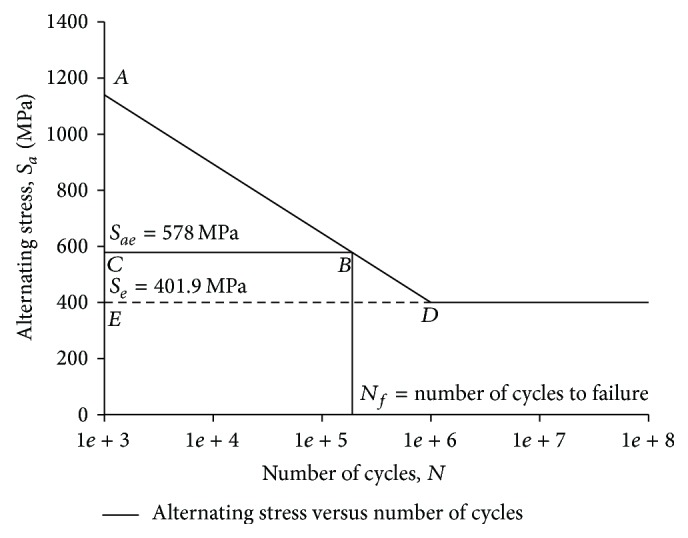
*S-N* curve for 65Si7.

Figure 7 shows the* S*-*N* diagram for 65Si7. Point A represents the alternating stress at which the spring will sustain 1000 cycles. Point D represents the endurance limit, that is, 401.9 MPa of 65Si7. The line CB represents the equivalent alternating stress. The intersection of alternating stress at point B will give the number of cycles to fatigue failure.

From Figure 7 *S-N* plot, it is observed that Δ*ABC is similar to *Δ*ADE*.

Hence, *AC*/*AE* = *CB*/*DE*

(2) $$\begin{array}{c} AC=\log A-\log C=0.296,\\ AE=\log A-\log E=0.454,\\ DE=\log D-\log E=\log10^{6}-\log10^{3}=3\\ CB=\frac{0.296\times 3}{0.454}=1.957\\ \text{Total}\;CB=3+1.97=4.9957\\ \text{Number}\;\text{of}\;\text{cycles}=10^{4.957}=90763\;\text{cycles}\;\text{to}\;\text{failure}. \end{array}$$

## 7. Analytical Method for Fatigue Life Prediction

**Figure 8:**
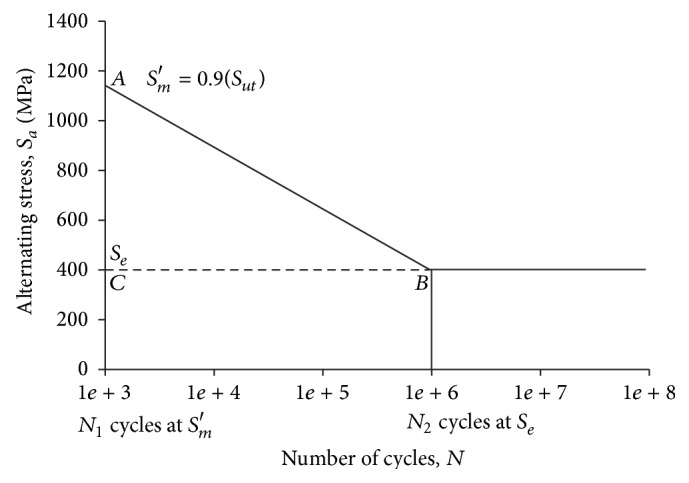
Estimated* S-N* curve.

The high cycle fatigue regime is from 10^3^ to 10^6^ and beyond. The material strength at 10^3^ cycles is called *S*_*m*_′, which is equal to 0.9 Sut. The estimated* S-N* diagram drawn on the log-log graph is shown in Figure 8. Figure 8 shows that the *x*-axis runs from 10^3^ to 10^9^ or beyond. The appropriate *S*_*m*_′ from the equation for bending load is plotted at *N*_1_ = 10^3^. As the material exhibits a knee, the corrected endurance limit *S*_*e*_ is plotted at *N*_2_ = 10^6^. A straight line is drawn between *S*_*m*_′ and *S*_*e*_. The curve is continuous horizontally beyond *N*_2_ point.

The equation of the line from *S*_*m*_′ to *S*_*e*_ can be written as

(2i) $$\begin{array}{c} S\left(N\right)=aN^{b}\;\left(\text{taking}\;\log\;\mathrm{on}\;\text{both}\;\text{sides}\right) \end{array}$$

(2i) $$\begin{array}{c} \log S\left(N\right)=\log a+b\;\log N, \end{array}$$

where *S*(*N*) is the fatigue strength at any *N* and *a*, *b* are the constants defined by the boundary conditions. For all the conditions, the *y* intercept is *S*(*N*) = *S*_*m*_′ at *N* = *N*_1_ = 10^3^, for endurance limit case *S*(*N*) = *S*_*e*_ at *N* = *N*_2_ = 10^6^. Applying the boundary condition in the equation, we have

(2i) $$\begin{array}{c} \log S_{m}^{\prime }=\log a+b\;\log N_{1} \end{array}$$

(2v) $$\begin{array}{c} \log S_{e}=\log a+b\;\log N_{2\;} \end{array}$$

(2v) $$\begin{array}{c} \log S_{m}^{\prime }-\log S_{e}=b(\log N_{1}-\log N_{2}) \end{array}$$

(2i) $$\begin{array}{c} Z=(\log N_{1}-\log N_{2}) \end{array}$$

(2i) $$\begin{array}{c} b=\frac{1}{Z}\log\frac{S_{m}^{\prime }}{S_{e}} \end{array}$$

(2i) $$\begin{array}{c} \log S_{m}^{\prime }-\;b\log N_{1}=\log a \end{array}$$

(2x) $$\begin{array}{c} \text{As}\;N_{1}=1000,\log_{10}1000=3 \end{array}$$

(2x) $$\begin{array}{c} N=\;{\left(\frac{S_{a}}{a}\right)}^{-1/b} \end{array}$$

**Figure 9:**
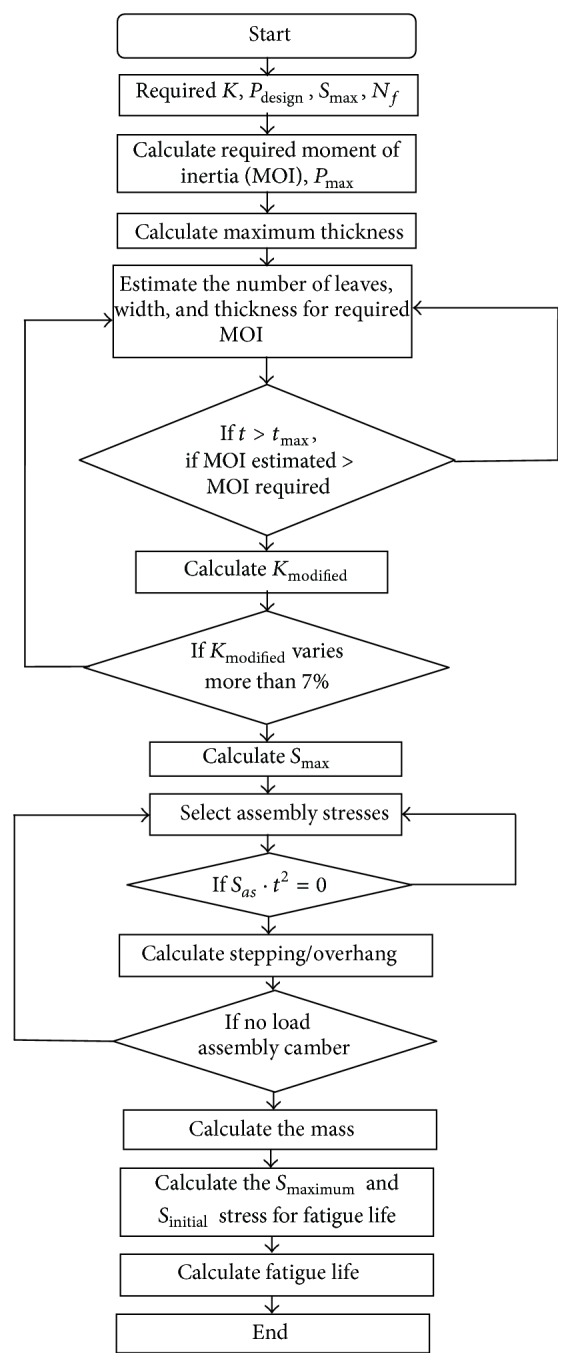
Flowchart for analytical estimation of fatigue life of 65Si7 leaf spring.

*S*_*ae*_ = *aN*_*f*_^*b*^ [13], where *N*_*f*_ = number of cycles to failure, *S*_*ae*_ = equivalent alternating stress, *a*, *b* are constants which depends on the material properties of 65Si7. The fatigue life is to be predicted at equivalent alternating stress so *S*_*a*_ is replaced by *S*_*ae*_. The above mentioned equation is used for fatigue life estimation and the codes for the analytical method are written in FORTRAN. The flowchart is shown in Figure 9.

## 8. Computer Aided Engineering Analysis

The CAD model of the individual leaf is prepared from the standard drawing of the leaf spring and assembled. The leaf spring assembly is imported to an analysis software ANSYS for further processing. The different contacts are set and entire model is meshed into small elements. The boundary conditions are applied by taking into consideration the actual loading conditions and the solver gives the solution. The diverse steps involved in CAE analysis are as follows.

### 8.1. CAD Modeling

**Figure 10:**
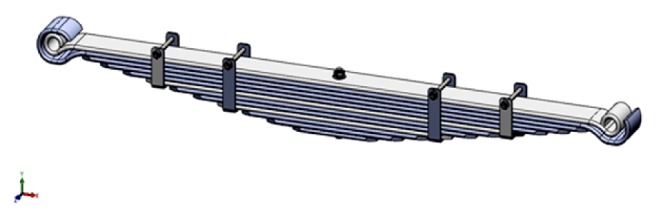
CAD model of the leaf springs.

The CAD modeling of the conventional leaf springs structure is performed by using solid works software. The CAD model of leaf springs consists of total 34 different parts which are assembled together in assembly design to construct an assembly. The multileaf springs assembly used for analysis is shown in Figure 10.

### 8.2. Analysis Using ANSYS

The CAD model of leaf springs has been imported in the ANSYS solver. All the boundary conditions and material properties have been specified as per the standard specified by the vehicle manufacturer. The material used for the leaf springs for analysis is 65Si7, which is having homogenous and isotropic behaviour. The material 65Si7 is added in the material library and the engineering properties are applied.

The process for performing analysis in ANSYS solver involves the following.

#### 8.2.1. Setting the Contact Reign

The contact conditions are formed where the components meet. The differences in the contact settings determine how the contacting bodies can move relative to one another. In this assembly, the No separation contact is used for the analysis. It only applies to region of faces. Separation of faces in contact is not allowed, but small amounts of frictionless sliding can occur along contact faces. In general, CONTA174 and TARGE170 are used. CONTA174 is used for contact and sliding between 3D target surfaces (TARGE170), which is defined by this element. The element is applicable to 3D structural analysis. TARGE170 is used to represent various 3D target surfaces for the associated contact elements.

#### 8.2.2. Meshing

In this congregation, SOLID187 element, which is a standard mechanical element for solids, is used for the result. SOLID187 element is a higher order 3-D, 10-node element. SOLID187 has quadratic displacement behaviour and is well suited to modeling irregular meshes. The element is defined by 10 nodes having three degrees of freedom at each node: translations in the nodal *x*, *y*, and *z* directions. The number of SOLID187 elements is 6964 and the number of nodes generated is 16943.

#### 8.2.3. Static Analysis Module

A static structural analysis determines the displacements, stresses, strains, and forces in structures or components caused by loads that do not induce significant inertia and damping effects. Steady loading and response conditions are assumed; that is, the loads and the structure's response are assumed to vary slowly with respect to time. Static structure analysis takes into consideration some parameters, like material properties, loading conditions, support conditions, and contacts which are to be specified as the input to the preprocessing of the analysis.

#### 8.2.4. Applying CAE Boundary Conditions

**Figure 11:**
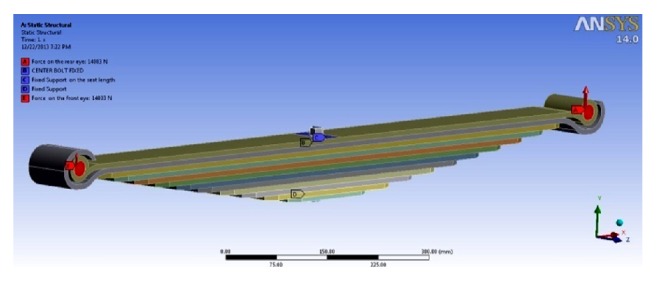
Boundary conditions for CAE analysis.

The boundary conditions are applied by taking into consideration the experimental loading conditions. The springs have been modelled in flat condition and the loading is done to achieve the initial condition. The total load is divided on the two eyes of the master leaf and pins. The fixed support constitutes the seat length (on master leaf and last (12th leaf) and the centre bolt. The CAE boundary conditions are shown in Figure 11.

### 8.3. Fatigue Life Assessment Using CAE Tools

While leaf springs may work well initially, they often fail in service due to fatigue failure caused by repeated cyclic loading. The aim of fatigue analysis is characterization of capability of a material to survive many cycles that a leaf spring may experience during its life time. In ansys fatigue module, two methods are available, that is, stress life and strain life method. For this work, the stress life method is used to predict the fatigue life of the component. The static environment is set by applying the boundary conditions by taking into consideration the actual loading conditions. After the static analysis, the fatigue tool is used for fatigue life estimation. In stress life decision tree, it is required to make four input decisions to perform a stress life analysis. These decisions affect the outcome of the fatigue analysis in both predicted life and types of postprocessing available. Input decisions that are to be considered for fatigue life estimation are loading type, mean stress effects, multiaxial stress correction, and fatigue modification factors.

#### 8.3.1. Type of Loading

Unlike static stress, which is analyzed with calculations for a single stress state, fatigue damage occurs when stress at a point changes over time. There are essentially four classes of fatigue loading, with the ANSYS fatigue module [14] currently supporting the first three:

- constant amplitude, proportional loading,
- constant amplitude, nonproportional loading,
- nonconstant amplitude, proportional loading,
- nonconstant amplitude, non-proportional loading.

Constant amplitude, proportional loading is the classic, “back of the envelope” calculation describing whether the load has a constant maximum value or continually varies with time. Loading is of constant amplitude because only one set of FE stress results along with a loading ratio is required to calculate the alternating and mean values. The loading ratio is defined as the ratio of the second load to the first load (LR = *L*_2_/*L*_1_). Loading is proportional since only one set of FE results are needed (principal stress axes do not change over time). Common types of constant amplitude loading are fully reversed (apply a load, and then apply an equal and opposite load; a load ratio of −1) and zero-based (apply a load and then remove it; a load ratio of 0). Since loading is proportional, looking at a single set of FE results can identify critical fatigue locations. Constant amplitude, nonproportional loading looks at exactly two load cases that need not to be related by a scale factor. The loading is of constant amplitude but nonproportional since principal stress or strain axes are free to change between the two load sets. Nonconstant amplitude, proportional loading also needs only one set of FE results. But instead of using a single load ratio to calculate alternating and mean values, the load ratio varies over time.

#### 8.3.2. Mean Stress Correction

For stress life, if experimental data at different mean stresses or r-ratio's exist, mean stress can be accounted for directly through interpolation between material curves. If experimental data is not available, several empirical options may be chosen including Gerber, Goodman, and Soderberg theories which use static material properties yield stress and tensile strength along with* S*-*N* data to account for any mean stress.

#### 8.3.3. Multiaxial Stress Correction Factor

Experimental test data is mostly uniaxial whereas finite element results are usually multiaxial. At some point, stress must be converted from a multiaxial stress state to a uniaxial one. Von-Mises stress, max shear stress, maximum principal stress, or any of the component stresses can be used for the comparison with the experimental uniaxial stress value.

#### 8.3.4. Fatigue Modifications (Value of Infinite Life)

Another available option when conducting a variable amplitude fatigue analysis is the ability to set the value used for infinite life. In constant amplitude loading, if the alternating stress is lower than the lowest alternating stress on the fatigue curve, the fatigue tool will use the life at the last point. This provides for an added level of safety because many materials do not exhibit an endurance limit.

**Figure 12:**
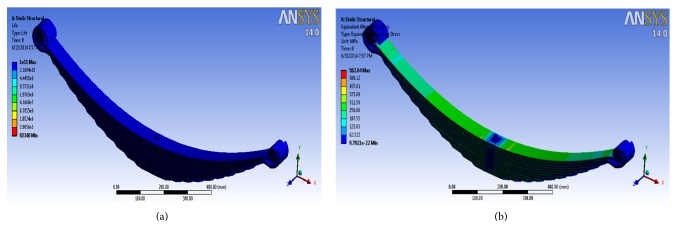
(a) Fatigue life using CAE tools. (b) Equivalent alternating stress.

The static environment is set and boundary conditions are applied, and the solver gives the solution. The deflection and stress results obtained from the specified loads are recorded in a tabular format. The CAE results for deflection and bending stress are shown in Table 5.  Figure 12(a) depicts the fatigue life of the leaf spring using Ansys solver. For prediction of fatigue life, the stress life approach is selected. A constant amplitude load ratio is selected as the loading type. The fatigue strength factor of 0.65 is chosen and scale factor is kept as 1. For mean stress theory, Goodman's criteria are selected. Lastly, for multiaxial stress correction, von-mises stress is selected and the component is designed for finite life. It has been observed that the fatigue life of the leaf spring using CAE tool is 82348 cycles.  Figure 12(b) depicts the equivalent alternating stress in the leaf spring using Ansys solver.

## 9. Results and Discussion

### 9.1. Fatigue Life Comparison by Various Approaches

Table 6 depicts the comparison of fatigue life by various methods. It is observed that, for the alternating stress level of 896–269 MPa, the experimental fatigue life of the leaf spring is 83513 cycles. The fatigue life estimated by SAE spring design manual technique is 69600 cycles, for the alternating stress level of 885–200 MPa. It is also observed that, in the SAE spring design manual approach, the maximum stress, that is, 885 MPa, is less than the specified maximum stress but the stress range is higher than specified stress range. Hence, this approximation provides results within 16.6% variation. The same approach with specified stress range gives the fatigue life of leaf spring to be 81600. For the same alternating stress level of 896–269 MPa, the fatigue life is found to be 90763 cycles by graphical method and equivalent stress is found to be 578 MPa. In the analytical method, the fatigue life is found to be 90304 cycles with 8.13% variation from the experimental results. The CAE fatigue life is found to be 82348 cycles, with 2.39% variation from the experimental results.

### 9.2. Comparison of Experimental and CAE Results

To validate the analysis, the CAE results have been compared with the experimental results. As the experiments are done on a full scale leaf spring testing machine under the specified loads, the CAE analysis has been carried out for the same loads. The maximum stress induced in the leaf springs is found to be 941 MPa and 989.89 MPa using experimental and CAE approach, respectively. The stress is found to be well below the yield tensile strength which is 1081.2 MPa. The total deformation and stress comparison for experimental and CAE approach also validates the CAE analysis of the leaf springs. The results of the comparison have been depicted in the tabular form in Table 7.

From Table 7, it is observed that, for the same unladen static load conditions, deflection in experimental and CAE results is 46.9 mm and 44.9 mm, respectively. The deformation observed in CAE results varies by 4.3%. The bending stress for experimental and CAE results is 262 MPa and 270.74 MPa, respectively. The variation of bending stress observed in CAE results from experimental is 3.3%. From Table 7, it is also observed that, for the same design load condition, the deflection found in experimental and CAE is 81.44 mm and 76.05 mm, respectively. The variation in CAE results from experimental is 6.6%. The bending stresses under the design load for experimental and CAE results are 446 MPa and 458 MPa, respectively. The variation in bending stress observed in the CAE results is 2.7%. For the maximum load condition, the static deflection is 176 mm and 164.36 mm for experimental and CAE results. It has been observed that when CAE results are compared with experimental, the variation in the deformation varies in between 4.3% and 6.6%, respectively. The stress variation lies in the range of 2.7% to 5.2%. It has also been observed that the maximum stress induced in the assembly in both the approaches is well below the yield stress of the material. It has been observed that the CAE results obtained by using SOLID187 mesh elements and CONTA172 and TARGE170 (No separation and sliding) contact elements provides the results closer to the experimental testing.

## 10. Conclusions

The 65Si7 LCV model is taken into consideration for determining the fatigue life by experimental and graphical methods, analytical method (using FORTRAN), SAE spring design manual approach, and CAE (using Ansys) approach. The following conclusions are made.

1. The fatigue life of the leaf spring for same stress range will decrease, if the magnitude of the initial stress is lower. In actual condition, the leaf springs will withstand 15–17% more fatigue life than predicted by SAE spring design manual approach.
2. Graphical and analytical methods are time consuming and prone to errors but provide results which are within 8-9% variation.
3. It is concluded that when CONTA174, TARGE170 type of contact and SOLID187 mesh element are used for CAE analysis, results are closer to the experimental results. CAE approach using Ansys predicts fatigue life within 2–5% of experimental results and can be an alternate of experimental and analytical approach.

## Figures and Tables

**Table 1:** Mechanical properties of 65Si7.

Mechanical property	Young's modulus, (*E*), MPa	BHN	Poisson's ratio, (*μ*)	Ultimate tensile strength, (*S*_*ut*_), MPa	Yield tensile strength, (*S*_*y*_), MPa	Elongation at fracture (minimum)	Density, (*ρ*), kg/mm^3^
Value	200124	380–432	0.266	1272	1081.2	7%	0.00000785

**Table 2:** Design parameters of the leaf springs.

Span, (*L*) (mm)	Load rate, (*k*) (N/mm)	Load (N)		No load camber, (*C*_*a*_) (mm)	Seat length (mm)	Total number of leaves (*N*)	Number of full length leaves (*X*)	Maximum thickness of the individual leaf (*t*) × width (*b*), (mm×mm)	Required fatigue life (*N*_*f*_) at (1.3 ± 0.7 g)
		Rated (*P*/*g*)	Maximum (*P*_max⁡_)						
1150 ± 3	159.11 ± 7%	12959	28010	95 ± 4	100	12	2	8 × 70	70000 cycles

**Table 3:** Experimental results for load, deflection, and bending stresses.

Serial number	Load type	Load (N)	Deflection (mm)	Bending stress (MPa)
1	Unladen load	7661	46.9	262
2	Design/rated load	12959	81.44	446
3	Flat load	15754	99	540
4	Rubber touching load	21645.7	136	743
5	Metal to metal contact	28010	176	941

**Table 4:** Experimental fatigue life of the specimen.

Material processing	Standard specified	Alternating stress level, (MPa)	Stress range	Fatigue life of S-1 (*N*_*f*_)	Fatigue life of S-2 (*N*_*f*_)	Fatigue life of S-3 (*N*_*f*_)	Fatigue life of S-4 (*N*_*f*_)	Average SD
Normal rolling, quenching at 880°C, hardening in oil at 80°C, tempering at 410°C for 90 mins, shot peening at 18 A intensity, BHN 380–432, and scragging at 0.9% of yield stress	0.6 g–2 g	269–896	627	84212	81961	82226	85656	Average 83513 SD 1518

**Table 5:** CAE results for load, deflection, and bending stresses.

Serial number	Load type	Load (N)	Deflection (mm)	Stress (MPa)
1	Unladen load	7661	44.9	270.74
2	Design/rated load	12959	76.05	458
3	Flat load	15754	92.45	556.83
4	Rubber touching load	21645.7	127.02	765
5	Metal to metal contact	28010	164.36	989.89

**Table 6:** Comparison of fatigue life by various approaches.

Serial number	Method for fatigue life assessment	Alternating stress level	Stress range	Equivalent alternating stress	Fatigue life	Variation from experimental results
1	Experimental	269–896	627	—	83513	—
2	SAE spring design manual approach	200–885	685	—	69600	16.6%
3	Graphical method	269–896	627	578	90763	8.68%
4	Analytical method	269–896	627	579	90304	8.13%
5	CAE method	269–896	627	562	82348	2.39%

**Table 7:** Comparison of experimental and CAE results.

Load type	Load (N)	Experimental results		CAE results		% age variation between CAE and Experimental results	
		Deflection	Stress	Deflection	Stress	Deflection	Stress
Unladen load	7661	46.9	262	44.9	270.74	4.3	3.3
Design/Rated load	12959	81.44	446	76.05	458	6.6	2.7
Flat load	15754	99	540	92.45	556.83	6.6	3.1
Rubber touching load	21645.7	136	743	127.02	765	6.6	3.0
Metal to metal contact load	28010	176	941	164.36	989.89	6.6	5.2

## Nomenclature

*C*_*a*_: No load assembly camber
*E*: Young's modulus of elasticity
*N*_*f*_: Number of cycles to failure
*ρ*: Density
*μ*: Poisson's ratio
*S*_*a*_: Alternating stress
*σ*_max⁡_: Maximum stress
*S*_*e*_′: Endurance limit
*S*_*e*_: Corrected endurance limit
*S*_*y*_: Yield tensile strength
*L*: Span
*σ*_min⁡_: Minimum stress
*S*_*m*_: Mean stress
*S*_*ae*_: Equivalent alternating stress
*S*_*ut*_: Ultimate tensile strength
*K*_surface_: Surface factor
*K*_load_: Load factor
*K*_temp_: Temperature factor
*K*_size_: Size factor
*K*_reliability_: : Reliability factor
*d*_equiv_: Equivalent diameter of rotating beam specimen for any cross section
*A*_95_: Portion of the cross sectional area of nonrounded that is stressed in between 95% and 100% of its maximum stress
*S*_*m*_′: Stress at 10^3^ cycles.

## References

[1] Saelem S., Chantranuwathana S., Panichanun K., Preedanood P., Wichienprakarn P., Kruo-ongarjnukool P. Experimental verification of leaf spring model by using a leaf spring test rig.

[2] Refngah F. N. A., Abdullah S., Jalar A., Chua L. B. (2009). Fatigue life evaluation of two types of steel leaf springs. *International Journal of Mechanical and Materials Engineering*.

[3] Fuentes J. J., Aguilar H. J., Rodríguez J. A., Herrera E. J. (2009). Premature fracture in automobile leaf springs. *Engineering Failure Analysis*.

[4] Aggarwal M. L., Khan R. A., Aggarwal V. P. (2006). Optimization of micro welds used in the leaf springs. *International Journal of Engineering Material and Science*.

[5] Aggarwal M. L., Agrawal V. P., Khan R. A. (2006). A stress approach model for predictions of fatigue life by shot peening of EN45A spring steel. *International Journal of Fatigue*.

[6] Patunkar M. M., Dolas D. R. (2011). Modelling and analysis of composite leaf spring under the static load condition by using FEA. *International Journal of Mechanical & Industrial Engineering*.

[7] Kumar M. S., Vijayarangan S. (2007). Analytical and experimental studies on fatigue life prediction of steel and composite multi-leaf spring for light passenger vehicles using life data analysis. *Materials Science*.

[8] Sanjurjo P., Rodríguez C., Pariente I. F., Belzunce F. J., Canteli A. F. (2010). The influence of shot peening on the fatigue behaviour of duplex stainless steels. *Procedia Engineering*.

[9] Zhuang W. Z., Halford G. R. (2001). Investigation of residual stress relaxation under cyclic load. *International Journal of Fatigue*.

[10] Savaidis G., Malikoutsakis M., Savaidis A. (2013). FE simulation of vehicle leaf spring behavior under driving manoeuvres. *International Journal of Structural Integrity*.

[11] IS 1135 (1995). *Springs—Leaf Springs Assembly for Automobiles*.

[12] SAE (1990). *Spring Design Manual—Design and Application of Leaf Springs*.

[13] Norton R. L. (2001). *Machine Design—An Integrated Approach*.

[14] Ansys Workbench training manual on fatigue tool.

